# Clinical simulation for nurses’ knowledge on postpartum hemorrhage: a randomized clinical trial

**DOI:** 10.1590/0034-7167-2024-0214

**Published:** 2025-03-14

**Authors:** Tatiane da Silva Coelho, Luciana Mara Monti Fonseca, Maria Vera Lúcia Moreira Leitão Cardoso, Priscila de Souza Aquino, Camila Chaves da Costa, Nathanael de Souza Maciel, Ana Kelve de Castro Damasceno

**Affiliations:** I Universidade Federal do Ceará. Fortaleza, Ceará, Brazil; II Universidade de São Paulo. Ribeirão Preto, São Paulo, Brazil; III Universidade da Integração Internacional da Lusofonia Afro-Brazileira. Redenção, Ceará, Brazil

**Keywords:** Nursing, Obstetric Nursing, Postpartum Hemorrhage, Education Nursing, Simulation Training, Enfermería, Enfermería Obstétrica, Hemorragia Posparto, Educación en Enfermería, Entrenamiento Simulado

## Abstract

**Objectives:**

to evaluate the effectiveness of a clinical simulation scenario in enhancing nurses’ knowledge of postpartum hemorrhage management.

**Methods:**

a randomized clinical trial was conducted in the obstetric center of a tertiary-level maternity hospital. Nurses involved in maternal care were divided into two groups. The control group received a didactic lecture (standard institutional training), while the intervention group, in addition to attending the lecture, participated in a clinical simulation for postpartum hemorrhage management. Data were analyzed using R software, version 4.2.0.

**Results:**

the sample consisted of 37 nurses, most of whom were female, with an average age of 40 years. The average pre-test score was 65%. After the clinical simulation-based intervention, the average post-test score increased to 90%.

**Conclusions:**

clinical simulation was effective in enhancing nurses’ knowledge of postpartum hemorrhage management.

## RESUMEN

**Objetivos:**

evaluar la eficacia de un escenario de simulación clínica para la adquisición de conocimientos de los enfermeros en el manejo de la hemorragia posparto.

**Métodos:**

ensayo clínico aleatorizado llevado a cabo en el centro obstétrico de una maternidad de nivel terciario. Los enfermeros que participan en la atención materna fueron divididos en dos grupos. El grupo de control recibió una clase expositiva dialogada (entrenamiento estándar de la institución), mientras que el grupo de intervención, además de recibir la clase, participó en la simulación clínica para el manejo de la hemorragia posparto. Los datos fueron analizados utilizando el software R 4.2.0.

**Resultados:**

la muestra estuvo compuesta por 37 enfermeros, la mayoría de sexo femenino, con una edad promedio de 40 años. El promedio de aciertos en el pretest fue del 65%. Tras la intervención basada en simulación clínica, el promedio de aciertos en el postest aumentó al 90%.

**Conclusiones:**

la utilización de la simulación clínica fue eficaz para la adquisición de conocimientos por parte de los enfermeros en el manejo de la hemorragia posparto.

## INTRODUCTION

Postpartum hemorrhage (PPH) is the leading cause of maternal death worldwide and one of the main causes of maternal mortality in the Americas, as well as peripartum hysterectomy. Reducing maternal mortality remains a challenge but is an achievable goal through joint and continuous efforts^(1,2)^. PPH is associated with approximately one-quarter of all maternal deaths globally and is the leading cause of maternal mortality in most low-income countries. It contributes to severe maternal morbidity and prolonged disabilities due to substantial blood loss, often progressing to shock and organ dysfunction^(2)^. In this context, reducing maternal mortality remains a challenge but is an achievable goal through ongoing and collaborative efforts.

One of the global political initiatives aimed at reducing maternal and child mortality is the Sustainable Development Goals (SDGs). Among these goals, reducing maternal mortality is specifically targeted in SDG 3.1: by 2030, reduce the global maternal mortality ratio to less than 70 deaths per 100,000 live births^(3)^. To make the SDGs more relevant to local challenges, the 2030 Agenda Goals were adapted to the national context, redefining SDG 3.1 to aim at reducing the maternal mortality ratio to a maximum of 30 deaths per 100,000 live births^(4)^.

For competent professionals to respond effectively to obstetric emergencies, early diagnosis, prevention, and treatment of PPH are essential in reducing maternal morbidity and mortality. Therefore, one of the strategies to improve care is to train healthcare teams to develop the necessary competencies for quality care, providing an objective and effective learning process that enhances patient safety. Training for PPH management can employ various strategies to acquire professional competence, one of which is clinical simulation, which facilitates the teaching-learning process^(5,6)^.

Clinical simulation in nursing education has been used to enhance competencies, improve communication among healthcare professionals, and evaluate the effectiveness of resources utilized in the simulation. The use of pre-designed and validated scenarios contributes to the success of simulation as a teaching strategy^(7-9)^.

It is well known that, in hospital organizations, the nursing team constitutes the largest workforce. Therefore, the success and effectiveness of simulations targeted at these professionals are linked to the positive outcomes obtained from transforming clinical practice^(9)^.

Clinical simulation in obstetric nursing has proven effective in providing care to vulnerable populations and contributes to reducing morbidity and mortality rates associated with preeclampsia, eclampsia, and other obstetric emergencies ^(7)^. The impact of acquiring competencies through clinical simulations is significant in reducing maternal mortality, as it ensures that all professionals attending women during childbirth have the opportunity to learn the necessary skills for managing PPH^(10)^.

In addressing nurses’ acquisition of knowledge about postpartum hemorrhage, the study recognizes the importance of ensuring adequate competencies to handle critical situations during childbirth, thereby contributing to the reduction of maternal mortality (SDG 3.1). Additionally, by using clinical simulation as an educational tool, the effectiveness of this method is highlighted in enhancing the practical skills of healthcare professionals, preparing them to face real challenges more efficiently and safely^(5,8)^. Thus, by integrating the training of nurses and other healthcare professionals with global health goals, the study demonstrates how education and training can play a fundamental role in promoting maternal health and achieving SDG.

## OBJECTIVES

To evaluate the effectiveness of a clinical simulation scenario in enhancing nurses’ knowledge in the management of PPH.

## METHODS

### Ethical Aspects

The ethical and legal principles outlined in Resolution No. 466/12 were followed. The study was submitted to and approved by the Research Ethics Committee of the Maternidade Escola Assis Chateaubriand, through the EBSERH Network and *Plataforma Brasil*. Written informed consent was obtained from all individuals involved in the study.

### Study Design, Period, and Location

This was a randomized controlled trial (RCT) with two parallel groups and a 1:1 allocation ratio, divided into five stages. The study followed the Consolidated Standards of Reporting Trials (CONSORT) for Randomized Trials of Nonpharmacologic Treatment^(11)^. It was registered in the Brazilian Clinical Trials Registry (ReBEC), RBR-6hk9vgs.

The study was conducted at the Maternidade Escola Assis Chateaubriand (MEAC), located in Fortaleza, Ceará, between October 2022 and March 2023. This institution is affiliated with the Federal University of Ceará and the Unified Health System (SUS). It is a tertiary reference maternity hospital for childbirth and newborn care, providing medium and high-complexity services in both hospital and outpatient settings for women and newborns. Additionally, it is part of the Zero Maternal Death from Hemorrhage (0MMxH) strategy.

### Population or Sample; Inclusion and Exclusion Criteria

The study population consisted of nurses of both genders who provided direct obstetric care to laboring and/or postpartum women in seven sectors of the maternity hospital: emergency, obstetric center, surgical center, post-anesthesia care unit (PACU), obstetric clinic, surgical clinic, and maternal intensive care unit (ICU).

The Control Group (CG) consisted of eligible nurses who attended a didactic lecture on the management of PPH, delivered by the principal investigator and sent via Google Forms link through WhatsApp, in addition to the routine training provided by the institution, which consists of on-the-job guidance through continuing education on PPH management. The Intervention Group (IG) consisted of eligible nurses who, in addition to participating in the didactic lecture on PPH management, also participated in the clinical simulation scenario for PPH management.

Given the specificity of nursing care, it was established that, to participate in the simulation, nurses were required to attend a lecture on PPH beforehand, have knowledge of the physiology and anatomy of labor, delivery, and the immediate postpartum period, be familiar with the treatment protocol for PPH, and understand biosafety resources and the materials used.

The sample selection followed these inclusion criteria: being a permanent nurse of the institution who provided direct obstetric care to laboring and/or postpartum women. The exclusion criteria were: having a self-declared compromised physical or mental health condition that would prevent participation in the clinical simulation. Nurses who were on vacation or reassigned to other sectors were also excluded. The discontinuity criteria were: dropping out of the study after the start of data collection; being unable or unwilling to participate in the clinical simulation; changing from a clinical to an administrative role, taking vacations, leaves, or breaking ties with the institution.

The sample was obtained using a sample size calculation based on the formula for comparing two experimental means with a bilateral test, considering the outcome as a quantitative variable in an unpaired sample^(12)^. Thus, the sample calculation considered a total of 32 participants, 16 in each group. Adding a 20% safety margin for potential losses, 20 nurses were required per group, totaling a sample size of 40 participants.

Recruitment occurred one month before the intervention in each sector. To reach this sample size, 73 nurses were invited to participate in the study, of whom 50 accepted the invitation. Eligible nurses received a copy of the Informed Consent Form (ICF) via Google Forms, delivered by the principal investigator, allowing time to read and understand the study details. Subsequently, those interested in the course filled out the registration form provided. The form included data such as name, phone number, email, and preliminary information about the nurse’s familiarity with the topic. The research objectives were presented to the participants.

Of those who accepted, only 41 signed the informed consent form and completed the sociodemographic questionnaire and pre-test (baseline). After completing the pre-test, the 41 nurses were randomly allocated to one of the groups using randomization software (randomized.org). Twenty-one nurses were allocated to the IG, with three lost to follow-up due to withdrawal or inability to participate. Twenty nurses were allocated to the CG, with one lost to follow-up due to withdrawal or inability to participate. Thus, 19 nurses comprised the CG, and 18 comprised the IG, making the final sample size 37 nurses.

### Study Intervention

The implemented intervention, the simulation scenario, was developed in 2016 and validated by 22 faculty members from the Federal University of Pernambuco (UFPE), including nurses with experience in clinical practice and/or teaching and research in women’s health/obstetrics, clinical simulation, or adult emergency care, for content validation. The selection of judges followed Fehring’s criteria, based on their curriculum and recommendations from other specialists^(7)^. The simulation scenario was conducted in the obstetric center.

In 2022, the scenario was updated and adapted according to the revised WHO protocol (2018) for PPH treatment. The updated scenario was validated again by 22 specialists and the target audience, maintaining the original theme and learning objectives.

The study was based on the simulation model proposed by Pamela Jeffries^(13)^, which provides a framework for designing, implementing, and evaluating simulations used as teaching strategies in nursing, as well as on the guidelines from the International Nursing Association for Clinical Simulation and Learning^(8)^. Recognizing the need for a consistent, empirically supported model to guide the design and implementation of simulations and evaluate outcomes, Pamela Jeffries developed a simulation framework that specifies relevant variables and their relationships, facilitating the identification of effective and ineffective practices in research.

This model for constructing simulation-based experiences establishes the following key elements distributed across eight chapters: simulation design, outcomes and objectives, facilitation, debriefing, participant evaluation, professional integrity, interprofessional education enhanced by simulation, and a simulation glossary. Best practices in health simulation include: initial and ongoing professional development, prebriefing, simulation design, facilitation, debriefing process, operations, outcomes and objectives, professional integrity, and the evaluation of learning and performance^(8)^.

The clinical simulation scenario was developed based on these recommendations, which guided the entire teaching-learning process. The first step involved disseminating information about the study across the seven sectors providing maternal care. After participants consented to join the study, a sociodemographic characterization instrument was administered to the nurses via a Google Form link shared in a WhatsApp group, collecting data on variables such as gender, age, education level, sector of employment, duration of employment in the sector, and specialty. Subsequently, the subjects were enrolled and randomly assigned to either the control or experimental group.

The knowledge assessment (pre-test) consisted of 15 multiple-choice questions related to PPH knowledge. Each question was worth 1 point, with a maximum score of 15 points. Additionally, for each question, the nurse indicated their level of confidence in their answer at the time of responding.

The knowledge assessment was developed by the researchers after conducting a literature review and examining protocols on the management of PPH (PAHO/WHO) and the most up-to-date institutional protocol available at the time of data collection. Following this stage, a pilot study was conducted to make adjustments for semantic accuracy and content clarity. No suggestions for modifications were reported.

The simulation scenario was designed to address the learning objectives related to a clinical case of a postpartum woman experiencing hemorrhage due to uterine atony. The scenario included the following aspects: 1) General and specific objectives; 2) Competencies and skills; 3) Materials; 4) Case description; 5) Guiding questions for debriefing; 6) Checklist of expected actions for monitoring practical performance. The checklist was created to assist researchers in evaluating the expected actions for PPH management and guiding the debriefing process.

The pilot study was conducted to identify and address potential gaps in the scenario. After testing the scenario and instruments, the simulation scenario was individually implemented with each volunteer nurse, lasting 45 minutes, with the following schedule: 5 minutes for prebriefing, 10 minutes for action, and 30 minutes for debriefing.

Before the participant began the simulated scenario, they participated in a handover conducted by a nurse who provided information about the case, including details of the delivery and the condition of the mother-baby dyad in the immediate postpartum period. To guide the simulated scenario, the checklist of expected actions outlined possible responses from evaluators, considering whether the actions were performed or not, correctly or incorrectly, and whether they were performed in a timely manner or delayed by the volunteer in the scene.

After the simulated scenario, the debriefing was conducted. The debriefing focused on discussions about the care provided to the postpartum woman experiencing hemorrhage and the simulated clinical condition. It was structured into emotional, descriptive, evaluative, analytical, and conclusive stages and was mediated by the facilitator, who initially encouraged verbal expressions from the participants involved in the scenario and subsequently from the other group members.

This moment allowed the volunteer nurse to reflect on the nursing care they provided, considering the assessment of the labor context, risk factors, clinical judgment, visual estimation of blood loss, shock index calculation, early diagnosis of PPH, calling for help, and initiating the medication protocol for PPH treatment. Additionally, positive points and areas for improvement were discussed.

### Analysis of results and statistics

Data were compiled and analyzed using R software, version 4.2.0. Measures of central tendency and dispersion were used. The Shapiro-Wilk test was applied to verify the assumption of normality. To compare the two groups, CG and IG, based on their scores on each instrument and other variables, the Wilcoxon test was used. The following hypotheses were formulated: the null hypothesis (H₀) states that there is no difference between clinical simulation and the traditional method in nurses’ knowledge of PPH management; the alternative hypothesis (H₁) suggests that there is a difference in nurses’ knowledge between the two methods. For this test, statistical significance was considered when p < 0.05.

## RESULTS

Of the 41 nurses randomized into the sample, 37 remained until the end of the study, with 18 in the IG and 19 in the CG. The distribution between professionals in the control and experimental groups was homogeneous. The average length of time working in the sector was 8 years, although the participants had been graduated for approximately 16 years.

The majority of the nurses were women, aged between 31 and 40 years, with a range of nursing education from 2 to 36 years since graduation, holding various levels of postgraduate qualifications, including specializations in diverse areas, as well as master’s and doctoral degrees (Table 1).

**Table 1:** Distribution of variables related to the characteristics of participants and profile of nurses working in prepartum, childbirth, and postpartum care sectors, Fortaleza, Ceará, Brazil, 2023

Sociodemographic variables	Control Group		Intervention Group		Total	
	n=19	%	n=18	%	N=37	%
Gender						
Female	18	48.7	11	29.7	29	78.4
Male	2	5.4	6	16.2	8	21.6
Age Range						
31-40 years	9	24.3	11	29.7	20	54.0
41-50 years	7	18.9	5	13.5	12	32.4
51-60 years	3	8.1	2	5.4	5	13.5
Education Level						
Undergraduate	---	---	1	2.7	1	2.7
Specialization	15	40.5	11	29.7	26	70.2
Master's	4	10.8	4	10.8	8	21.6
Doctorate	---	---	2	5.4	2	5.4
OB Nursing Specialization						
Yes	8	21.6	11	29.7	19	51.3
No	11	29.7	7	18.9	18	48.6
Work Sector						
Emergency	2	5.4	5	13.5	7	18.9
Maternal ICU	2	5.4	---	---	2	5.4
Surgical Center	4	10.8	2	5.4	6	16.2
Surgical Clinic	2	5.4	1	2.7	3	8.1
Obstetric Clinic	4	10.8	3	8.1	7	18.9
Obstetric Center	5	13.5	5	13.5	10	27.1
PACU	---	---	2	5.4	2	5.4
Time working in the sector						
< 6 months	---	---	2	5.4	2	5.4
1-10 years	15	40.5	13	35.1	28	75.6
11-20 years	3	8.1	3	8.1	6	16.2
21-30 years	1	2.7	---	---	1	2.7
Total	19	51.3	18	48.7	37	100

*OB - Obstetric; ICU - Intensive Care Unit; PACU - Post-Anesthesia Care Unit.*

At baseline, most nurses had at least some prior experience with simulation; over 80% of the nurses had received some type of prior training for the management of PPH, and almost all participants had this opportunity provided by their employing institution. For most of them, the training for PPH management had been conducted more than a year prior. Approximately 91% of the nurses reported having previously assisted a postpartum woman with hemorrhage; however, about 30% of the nurses stated that they did not feel prepared to manage PPH due to fear/insecurity or lack of training/knowledge (Table 2).

**Table 2:** Distribution of variables related to nurses’ participation in activities involving simulation and training for postpartum hemorrhage management, Fortaleza, Ceará, Brazil, 2023

Activities involving simulation and training for PPH management	Control Group		Intervention Group		Total	
	n=19	%	n=18	%	N=37	%
Previous experience with simulation (n=37)						
Yes	10	27.0	11	29.7	21	56.7
No	9	24.3	7	19.0	16	43.3
Previous training for PPH management (n=37)						
Yes	15	40.5	16	43.3	31	83.8
No	4	10.8	2	5.4	6	16.2
In what context (n=31)						
Course provided by the institution	14	45.2	16	51.6	30	96.8
In various contexts	1	3.2	---	---	1	3.2
Time since last training (n=31)						
<1 year	2	6.5	4	13.0	6	19.5
1-2 years	11	35.5	7	22.5	18	58.0
>2 years	2	6.4	5	16.1	7	22.5
Has managed a PPH before (n=37)						
Yes	18	48.6	16	43.3	34	91.9
No	1	2.7	2	5.4	3	8.1
In what context (n=34)						
In advanced training courses	2	5.9	2	5.9	4	11.8
During shifts	15	44.1	13	38.2	28	82.3
In various contexts						
Feels prepared for PPH management (n=37)						
Yes	15	40.5	11	29.7	26	70.2
No	4	10.8	7	19.0	11	29.8
If not. what are the reasons (n=11)						
Fear and/or insecurity	2	18.2	2	18.2	4	36.4
Lack of trained staff/knowledge	2	18.2	5	45.4	7	63.6
Total	19	51.3	18	48.7	37	100

*PPH - Postpartum Hemorrhage.*

Regarding the comparison between the number of correct answers after the intervention and the clinical competence of the nurses, statistical significance was observed (p<0.00), demonstrating an association between the level of prior competence and the cognitive performance of participants after the simulation scenario. This indicates an increase/improvement in knowledge regarding the management of PPH, with a greater than 60% increase in correct answers in the post-test.

There was an improvement in the number of correct answers before and after the intervention. In the pre-test, among the 15 objective questions, the average number of correct answers was 3.8, with a range of 1-9 correct answers, and no nurse scored more than 60% on the pre-test questions. In the post-test, out of the 15 questions presented, the average number of correct answers was 13.24, with a range of 9-15 correct answers, with a minimum of 60% and a maximum of 100% cognitive performance. A statistically significant result was observed (p = 0.00) for the post-intervention results (Table 3).

**Table 3:** Distribution of the number of correct answers in the pre-test and the number of correct answers in the post-test. Results before and after the intervention, Fortaleza, Ceará, Brazil, 2023

Answers		Correct answers pre-test	Correct answers Post-test	
1	Regarding the possible causes of PPH	1/37	23/37	0.00^*^
2	About early treatment of PPH	0/37	36/37	
3	About risk stratification for PPH	4/37	31/37	
4	Initial assessment of a patient with PPH	13/37	36/37	
5	Regarding the golden hour in PPH	8/37	32/37	
6	Related to the Shock Index (SI)	5/37	34/37	
7	About the visual diagnosis of blood loss in PPH	1/37	27/37	
8	About the standard prescription for PPH management	6/37	29/37	
9	About teamwork in PPH management	11/37	37/37	
10	About non-surgical management of PPH	10/37	31/37	
11	Regarding preventive measures for PPH	6/37	36/37	
12	About the clinical method for diagnosing PPH	2/37	36/37	
13	Nurses’ competencies in managing a PPH case	15/37	36/37	
14	About the PPH material/kit	7/37	36/37	
15	Definition of massive PPH	4/37	30/37	

* *Wilcoxon test . PPH - Postpartum Hemorrhage.*

The results of the nurses in the CG, who were exposed to the didactic lecture and the training typically offered by the institution, showed a 57.4% improvement in cognitive performance regarding knowledge of PPH management before and after the intervention. For the IG, who were exposed to the didactic lecture and the clinical simulation scenario, an improvement in cognitive performance of 67.8% was observed.

Table 4 presents the measures of central tendency (mean and median) and dispersion (standard deviation) of the pre-test and post-test scores for each group. In addition to the intergroup comparative analysis before and after the intervention, a statistically significant difference was observed (p < 0.00), rejecting the null hypothesis and indicating that clinical simulation has a significant effect on the knowledge of nursing professionals compared to the traditional method.

**Table 4:** Measures related to knowledge before (pre-test) and after (post-test) between the control and intervention groups, Fortaleza, Ceará, Brazil, 2023

Grupos		Mean	Median	SD	*p* value
Pre-test	Control Group	4.05	4	1.87	0.00^*^
	Intervention Group	3.72	3	2.22	
Post-test	Control Group	12.6	13	1.68	
	Intervention Group	13.9	14	1.21	

SD - standard deviation;

* Wilcoxon test.

## DISCUSSION

Regarding baseline results, previous experience with simulation showed that most nurses reported having some form of contact with simulation-based learning. Indeed, simulated learning experiences can provide participants with the development of specific clinical skills, as well as support decision-making and the development of critical thinking skills in a safe and controlled environment through simulated learning scenarios^(13)^. The interactive and multisensory nature of a simulated learning scenario creates an environment in which participants have the opportunity to receive immediate feedback from the facilitator regarding their performance^(14)^.

Regarding this tool for integrating theory and practice, clinical simulation offers contributions such as: the possibility of training prior to contact with real patients; opportunities for the development and application of knowledge and skills in a safe environment; minimization of errors through repetition and extensive training by the student; increased perception of responsibility toward care; valuable experience for psychomotor learning and the development of critical-reflective thinking; increased confidence and communication skills; and the expansion of contextualized learning that spans the entirety of care^(15)^.

The use of simulation as a tool for the teaching-learning process, in studies conducted in the United States and India, has shown that the use of simulators for team training can provide a reference for fostering interprofessional cooperation, improving clinical performance in PPH management, enhancing communication, and adherence to protocols among participants during simulated PPH scenarios, contributing to the reduction of morbidity from this obstetric emergency^(10,16)^.

The knowledge of the nurses examined, in both the control and experimental groups, was similar in the pre-test and showed improvement in the post-intervention evaluation. Along with the described data, an intervention study of the before-and-after type, conducted with 46 nursing students, corroborated the results presented here, demonstrating that learning through clinical simulation resulted in higher knowledge scores compared to those acquired through didactic lectures with skill demonstrations^(17)^. In line with our findings, meta-analysis studies have shown that simulation-based nursing education is an effective method in the teaching-learning process^(18)^.

The improvement in cognitive performance promoted by the use of clinical simulation stands out, presenting potential positive impacts on nurses’ learning and clinical practice. The great potential of simulation to address issues related to the development of knowledge and skills among professionals is evident, as it surpasses the passive reception process centered on content transmission present in traditional education^(19)^.

Both groups expressed that there are other issues related to meaningful learning that may be interfering with the results and preventing nurses from feeling prepared to care for a postpartum woman with hemorrhage. As revealed by an integrative review aimed at synthesizing scientific production on the Theory of Meaningful Learning in the nursing teaching-learning process, the authors concluded that it is necessary to break the dichotomy between theory and practice and promote the articulation of content with action, introducing the student as the author of their own knowledge^(20)^.

A descriptive and theoretical study with a qualitative approach reported that integrating the Theory of Meaningful Learning and Andragogy is possible, especially when dealing with adult learners. However, before attempting to implement this integration, facilitator-teachers must move away from the role of mere knowledge transmitters, and students must abandon the role of passive receivers. This is a difficult exercise but one that must be practiced continuously, as only then will teaching and learning activities be transformed into moments of satisfaction, leading to greater achievement of the proposed objectives^(20)^.

Regarding experiential practice, the results of a qualitative and descriptive study conducted with students of a specialization course in obstetric nursing revealed that using high-fidelity human patient simulators can be employed in complex case scenarios to promote experiential learning in clinical emergencies^(14)^.

Additionally, in the context of clinical practice, implementing educational approaches that consider practical experiences has substantial value, as professionals build their own cognitive structures and consolidate knowledge through interaction with their environment^(21)^.

The execution of specific skills is fundamental, but it is through the resolution of complex scenarios in simulated contexts that nurses consolidate their knowledge and develop critical thinking, judgment, decision-making abilities, and competencies across different technical, attitudinal, and ethical dimensions^(22)^.

Benner, in her latest work, calls for a radical transformation in nursing education, where students should develop a fluid relationship between scientific knowledge, qualified know-how, and ethics, enriching the curriculum with experiential learning. Experiential learning should occur not only in the clinical setting but also in the classroom and skills laboratory. This learning should help students develop a sense of relevance, clinical reasoning, and clinical imagination while forming their identities as nurses^(23)^.

### Study limitations

The study had several limitations, including the inability to evaluate long-term knowledge retention since the results were not monitored over time, and the fact that the study was conducted at a single institution. Additionally, there was a reduced sample size due to the difficulty of nurse participation, as they had to balance their workload with the educational intervention, and due to sample losses. The study was also limited to a single setting, the obstetric center.

### Contributions to the Field

PPH is a highly preventable cause of maternal morbidity and mortality worldwide. Efforts to improve the diagnosis and management of PPH may include clinical simulation. The study’s contribution relates to the potential enhancement of meaningful learning in the management of PPH, alongside the acquisition of competencies by nurses and healthcare professionals. This research contributes to the scientific advancement of studies on nursing simulation through the use of scenarios.

Thus, the study presents a powerful tool for improving the quality of care in this obstetric emergency and highlights a situation that, unfortunately, continues to claim the lives of many women and requires decisive action by nurses to change this reality.

## CONCLUSIONS

The educational intervention using clinical simulation as a teaching-learning strategy proved effective in enhancing nurses’ knowledge of PPH management, thereby improving this knowledge, which is essential for achieving SDG 3.1 and contributing to the reduction of maternal mortality. The results showed a significant impact on the cognitive performance of nurses, regardless of their education level and previous experience, as demonstrated by the evaluation after the simulation activity compared to the pre-intervention period.
