# Supplementary material for: Clinical simulation for nurses’ knowledge on postpartum hemorrhage: a randomized clinical trial
Source: Rev Bras Enferm. 2025 Mar 14;78(Suppl 1):e20240214. doi: 10.1590/0034-7167-2024-0214 (PMC11913133; doi:10.1590/0034-7167-2024-0214)
Supplement: Translation

# Translation: Simulação clínica para o conhecimento de enfermeiros sobre hemorragia pós-parto: ensaio clínico randomizado

**DOI:** 10.1590/0034-7167-2024-0214pt

**Authors:** Tatiane da Silva Coelho, Luciana Mara Monti Fonseca, Maria Vera Lúcia Moreira Leitão Cardoso, Priscila de Souza Aquino, Camila Chaves da Costa, Nathanael de Souza Maciel, Ana Kelve de Castro Damasceno

**Affiliations:** I Universidade Federal do Ceará. Fortaleza, Ceará, Brasil; II Universidade de São Paulo. Ribeirão Preto, São Paulo, Brasil; III Universidade da Integração Internacional da Lusofonia Afro-Brasileira. Redenção, Ceará, Brasil

**Keywords:** Enfermagem, Enfermagem Obstétrica, Hemorragia Pós-Parto, Educação em Enfermagem, Treinamento por Simulação

## Abstract

**Objetivos:**

avaliar a eficácia de um cenário de simulação clínica para a aquisição de conhecimentos de enfermeiros no manejo da hemorragia pós-parto.

**Métodos:**

ensaio clínico randomizado conduzido no centro obstétrico de uma maternidade de nível terciário. Enfermeiros atuantes na assistência materna foram divididos em dois grupos. O grupo controle recebeu uma aula expositiva dialogada (treinamento padrão da instituição), enquanto o grupo de intervenção, além de receber a aula, participou da simulação clínica para o manejo da hemorragia pós-parto. Os dados foram analisados usando o software R 4.2.0.

**Resultados:**

a amostra foi constituída por 37 enfermeiros, a maioria do sexo feminino, com média de idade de 40 anos. A média de acertos no pré-teste foi de 65%. Após a intervenção baseada em simulação clínica, a média de acertos no pós-teste aumentou para 90%.

**Conclusões:**

a utilização da simulação clínica foi eficaz para a aquisição de conhecimento pelos enfermeiros no manejo da hemorragia pós-parto.

## INTRODUÇÃO

A hemorragia pós-parto (HPP) é a maior causa mundial de morte materna e uma das principais causas de mortes de mães na região das Américas e de histerectomia periparto. A busca pela redução da mortalidade materna continua sendo um desafio, mas é uma meta alcançável por meio de esforços conjuntos e contínuos^(1,2)^. A HPP está associada a aproximadamente um quarto de todas as mortes maternas no mundo e, na maioria dos países de baixa renda, é a principal causa de mortalidade materna. Ela contribui para a morbidade materna grave e para incapacidades prolongadas devido à perda substancial de sangue, com evolução para choque e disfunção orgânica^(2)^. Nesse sentido, a redução da mortalidade materna permanece um desafio, mas é uma meta alcançável por meio de esforços conjuntos e contínuos.

Um dos enfrentamentos políticos mundiais que visam a redução da mortalidade materna e infantil são os Objetivos de Desenvolvimento Sustentável (ODS). Dentre estes objetivos, a redução da mortalidade materna é contemplada na Meta ODS 3.1: até 2030, reduzir a taxa de mortalidade materna global para menos de 70 mortes por 100.000 nascidos vivos^(3)^. Com vistas a tornar os ODS mais pertinentes aos desafios locais, as Metas da Agenda 2030 foram adaptadas à realidade nacional, redefinindo o ODS 3.1 para reduzir a razão de mortalidade materna para, no máximo, 30 mortes por 100 mil nascidos vivos^(4)^.

Para a atuação de profissionais competentes na assistência às emergências obstétricas, o diagnóstico precoce, a prevenção e o tratamento da HPP são fundamentais para a redução da morbimortalidade materna. Portanto, uma das estratégias para melhorar a assistência é o treinamento da equipe de saúde, com o objetivo de desenvolver as competências necessárias para uma assistência de qualidade, proporcionando um processo de aprendizagem de forma objetiva e efetiva, e possibilitando uma segurança adequada ao paciente. O treinamento para o manejo da HPP pode utilizar diversas estratégias para a aquisição da competência profissional, sendo uma delas a simulação clínica, que facilita o processo de ensino-aprendizagem^(5,6)^.

A simulação clínica no ensino da enfermagem tem sido utilizada para aumentar as competências, melhorar a comunicação entre os profissionais de saúde e avaliar a eficácia dos recursos utilizados na simulação. A utilização de cenários previamente elaborados e validados colabora com o sucesso da simulação como estratégia de ensino^(7-9)^.

Sabe-se que, nas organizações hospitalares, a equipe de enfermagem constitui o maior contingente de trabalhadores. Portanto, o sucesso e a efetividade de simulações direcionadas a esses profissionais estão relacionados aos efeitos positivos obtidos a partir da transformação da prática laboral^(9)^.

A simulação clínica na Enfermagem Obstétrica mostrou-se eficaz durante a prestação de cuidados à população vulnerável e contribui para a redução das taxas de morbidade e mortalidade associadas à pré-eclâmpsia, eclâmpsia e outras emergências obstétricas^(7)^. O impacto da aquisição de competências por meio de simulações clínicas é significativo para reduzir a mortalidade materna, pois assegura que todos os profissionais que atendem as mulheres durante o parto tenham a oportunidade de aprender as competências necessárias para o gerenciamento da HPP^(10)^.

Ao abordar a aquisição de conhecimentos dos enfermeiros sobre HPP, o estudo reconhece a relevância de garantir competências adequadas para lidar com situações críticas durante o parto, contribuindo assim para a redução da mortalidade materna (ODS 3.1). Além disso, ao utilizar a simulação clínica como ferramenta educacional, ressalta-se a eficácia desse método no aprimoramento das habilidades práticas dos profissionais de saúde, preparando-os para enfrentar desafios reais de forma mais eficiente e segura^(5,8)^. Assim, ao integrar a formação dos enfermeiros, bem como dos demais profissionais de saúde, com os objetivos de saúde global, o estudo demonstra como a educação e a capacitação podem desempenhar um papel fundamental na promoção da saúde materna e no alcance das metas de desenvolvimento sustentável.

## OBJETIVOS

Avaliar a eficácia de um cenário de simulação clínica para a aquisição de conhecimentos dos enfermeiros no manejo da HPP.

## MÉTODOS

### Aspectos éticos

Foram cumpridos os preceitos éticos e legais contidos na Resolução Nº 466/12. O estudo foi submetido e aprovado pelo Comitê de Ética em Pesquisa da Maternidade Escola Assis Chateaubriand, por meio da Rede EBSERH e da Plataforma Brasil. O Consentimento Livre e Esclarecido foi obtido de todos os indivíduos envolvidos no estudo, por escrito.

### Desenho, período e local do estudo

Trata-se de um ensaio clínico controlado randomizado (ECR), com dois grupos paralelos e taxa de alocação 1:1, dividido em cinco etapas. Além disso, seguiu-se o *Consolidated Standards of Reporting Trials* (CONSORT) for *Randomized Trials of Nonpharmacologic Treatment* ^(11)^. O estudo foi registrado na base de dados de Registro de Ensaios Clínicos Brasileiros (ReBEC), RBR-6hk9vgs.

O estudo foi desenvolvido na Maternidade Escola Assis Chateaubriand (MEAC), localizada na cidade de Fortaleza, no estado do Ceará, entre os meses de outubro de 2022 a março de 2023. Essa instituição é vinculada à Universidade Federal do Ceará e ao Sistema Único de Saúde (SUS). É uma maternidade de referência terciária para assistência ao parto e nascimento, sendo desenvolvidas atividades de média e alta complexidade na atenção hospitalar e ambulatorial à mulher e ao recém-nascido. Além disso, também compõe a estratégia Zero Morte Materna por Hemorragia (0MMxH).

### População ou amostra; critérios de inclusão e exclusão

A população do estudo foi constituída por enfermeiros, de ambos os sexos, que prestam assistência obstétrica direta às parturientes e/ou puérperas em sete setores que prestam assistência materna na maternidade: emergência, centro obstétrico, centro cirúrgico, sala de recuperação pós-anestésica (SRPA), clínica obstétrica, clínica cirúrgica e unidade de terapia intensiva (UTI) materna.

O Grupo Controle (GC) foi composto por enfermeiros elegíveis que assistiram a uma aula expositiva dialogada sobre o manejo da HPP, ministrada pela pesquisadora principal e enviada via link do Google Formulário pelo WhatsApp, além do treinamento comumente disponibilizado pela instituição, que consiste na capacitação de rotina para o manejo da HPP, ou seja, orientações realizadas em serviço pela educação permanente. O Grupo Intervenção (GI) foi composto por enfermeiros elegíveis que, além de participar da aula expositiva dialogada sobre o manejo da HPP, participaram do cenário de simulação clínica para o manejo da HPP.

Por se tratar de um cuidado de enfermagem específico, estabeleceu-se que, para a participação na simulação, os profissionais de enfermagem deveriam assistir previamente a uma aula sobre HPP, ter conhecimento da fisiologia e anatomia do trabalho de parto, parto e puerpério imediato, leitura do protocolo de tratamento para HPP, e conhecimento dos recursos de biossegurança e dos materiais utilizados.

A seleção da amostra obedeceu aos seguintes critérios de inclusão: ser enfermeiro do quadro permanente da instituição que atuasse na assistência obstétrica direta às parturientes e/ou puérperas. Os critérios de exclusão foram: apresentar estado de saúde físico ou mental comprometido, autodeclarado pelo participante, de modo a inviabilizar a realização da simulação clínica. Aqueles que estivessem de férias e/ou remanejados de setor foram excluídos do estudo. Os critérios de descontinuidade foram: desistir de participar do estudo após o início da coleta; desistir ou ser impossibilitado de acompanhar a simulação clínica; mudança de função assistencial para administrativa, férias, licenças e/ou quebra de vínculo com a instituição.

A amostra foi obtida por meio do cálculo amostral, realizado utilizando-se a fórmula para grupos comparativos de duas médias experimentais com teste bilateral, considerando como desfecho variável quantitativa em amostra não pareada^(12)^. Dessa forma, para o cálculo amostral foi considerado um total de 32 participantes, sendo 16 para cada grupo. Adicionando-se a esse total um percentual de segurança de 20% para eventuais perdas, foram necessários 20 enfermeiros para cada grupo, totalizando um tamanho amostral de 40 participantes.

O recrutamento ocorreu um mês antes do início da intervenção, em cada um dos setores. Para atingir essa amostra, 73 enfermeiros foram convidados para participar do estudo, dos quais 50 aceitaram o convite. Os enfermeiros elegíveis receberam uma cópia do Termo de Consentimento Livre e Esclarecido (TCLE), via formulário do Google, entregue pela pesquisadora principal, para que tivessem tempo de ler e se informar acerca do estudo. Posteriormente, os interessados no curso preencheram a ficha de inscrição disponibilizada. A ficha continha dados referentes a nome, telefone, e-mail e algumas informações preliminares sobre a proximidade do enfermeiro com a temática abordada. Os objetivos da pesquisa foram apresentados aos participantes.

Do total de aceites, apenas 41 assinaram o termo de consentimento livre e esclarecido e responderam ao questionário sociodemográfico e ao pré-teste (linha de base). Após responderem ao pré-teste, os 41 enfermeiros foram alocados, aleatoriamente, por meio de um *software* de sorteio (*randomized.org*), para um dos grupos. Foram alocados 21 enfermeiros para o GI, havendo 3 perdas de seguimento por desistência ou impossibilidade de participação. Para o GC, foram alocados 20 enfermeiros, havendo uma perda de seguimento por desistência ou impossibilidade de participação. Assim, 19 compuseram o GC e 18, o GI, sendo a amostra final de 37 enfermeiros.

### Intervenção do estudo

A intervenção implementada, o cenário de simulação, foi construída em 2016 e validada por 22 docentes da Universidade Federal de Pernambuco (UFPE), incluindo enfermeiros com experiência assistencial e/ou em docência e pesquisa nas áreas de saúde da mulher/obstetrícia e/ou simulação clínica ou emergência com adultos, para validação do conteúdo. A escolha dos juízes seguiu os critérios de Fehring, baseando-se no currículo e na indicação de outros especialistas^(7)^. Enfatiza-se que o cenário do estudo (simulação clínica) foi realizado no centro obstétrico.

No ano de 2022, o cenário passou por uma atualização e adaptação devido à atualização do protocolo da Organização Mundial de Saúde (OMS) (2018) para tratamento da HPP. O cenário atualizado e adaptado foi validado novamente por 22 especialistas e com o público-alvo, mantendo a temática e os objetivos de aprendizagem.

O estudo foi baseado no modelo de simulação proposto por Pâmela Jeffries^(13)^, que oferece uma estrutura para projetar, implementar e avaliar simulações utilizadas como estratégias de ensino na enfermagem, bem como nos guias da I*nternational Nursing Association for Clinical Simulation and Learning* ^(8)^. Com a necessidade de um modelo consistente e com suporte empírico para orientar o desenho e implementação de simulações e avaliar os resultados, incitado por educadores de enfermagem, Pâmela Jeffries criou uma estrutura de simulação que especifica variáveis relevantes e suas relações para conduzir pesquisa de forma organizada, facilitando a identificação de práticas eficazes e ineficazes.

Esse modelo para construção de experiências baseadas em simulação estabelece os seguintes elementos-chave distribuídos em oito capítulos: design da simulação, resultados e objetivos, facilitação, *debriefing*, avaliação do participante, integridade profissional, educação interprofissional aprimorada por simulação e glossário de simulação. Os padrões de boas práticas em simulação em saúde incluem: Desenvolvimento profissional inicial e contínuo, *Prebriefing*, Projeto de simulação, Facilitação, Processo de *Debriefing*, Operações, Resultados e objetivos, Integridade Profissional e Avaliação de Aprendizagem e Desempenho^(8)^.

O cenário de simulação clínica foi elaborado com base nessas recomendações, que guiaram toda a formulação do processo de ensino-aprendizagem. A primeira etapa compreendeu a divulgação do estudo nos sete setores que prestam assistência materna. Após o aceite de participação na pesquisa, foi aplicado um instrumento de caracterização sociodemográfica dos enfermeiros, enviado por meio de link do *Google* Formulário no grupo de *WhatsApp*, com as seguintes variáveis: sexo, idade, tempo e nível de formação, setor de atuação, tempo de atuação no referido setor e especialidade. Posteriormente, o arrolamento dos sujeitos foi realizado, e os enfermeiros foram distribuídos, através de sorteio, no GC ou no experimental.

A avaliação de conhecimento (pré-teste) possuía 15 questões de múltipla escolha referentes aos conhecimentos sobre HPP. Cada questão valia 1 ponto, sendo considerado o máximo de acertos o enfermeiro que obtivesse 15 pontos. Além disso, para cada questão, o enfermeiro assinalou a opção que definia o nível de certeza que ele possuía no momento da resolução da questão.

A avaliação de conhecimento foi construída pelos pesquisadores após a realização de levantamento da literatura e dos protocolos sobre o manejo da HPP (OPAS/OMS) e do protocolo institucional mais atualizados até a data de coleta de dados. Após essa etapa, procedeu-se à aplicação do estudo piloto para realizar ajustes de semântica e clareza de conteúdo. Não foram apontadas sugestões de modificações.

O cenário de simulação apresentou o objetivo de aprendizagem relacionado ao caso clínico de uma puérpera com HPP, causada por atonia uterina. O cenário continha os seguintes aspectos: 1) Objetivo geral e específico; 2) Competências e habilidades; 3) Materiais; 4) Descrição do caso; 5) Questões norteadoras para o *debriefing*; 6) Checklist de ações esperadas para o acompanhamento de performance prática. Ressalta-se que o checklist de ações esperadas foi criado com o intuito de auxiliar os pesquisadores na avaliação das ações esperadas para o manejo da HPP e para a condução do *debriefing*.

O estudo piloto foi executado para sanar possíveis lacunas do cenário. Após a testagem do cenário e dos instrumentos, foi implementado o cenário de simulação, individualmente, com cada enfermeiro voluntário, com duração de 45 minutos, com a seguinte programação: 5 minutos para o *prebriefing*, 10 minutos para a ação e 30 minutos para o *debriefing*.

Antes do início da ação do participante na cena simulada, eles participaram da passagem de plantão por um enfermeiro, que transmitiu as informações referentes aos dados do caso, como ocorreu o parto e como estava o binômio mãe-bebê naquele momento de pós-parto imediato. Para o desenvolvimento do cenário simulado, o checklist de ações esperadas traz as possibilidades de respostas dos avaliadores, considerando a realização ou não, correta ou incorreta, e a realização no tempo adequado ou tardiamente pelo voluntário em cena.

Após a cena simulada, foi realizado o *debriefing*. O *debriefing* foi conduzido com foco nas discussões relativas aos cuidados prestados à puérpera com HPP e ao quadro clínico simulado. Estruturado seguindo as etapas emocional, descritiva, avaliativa, analítica e conclusiva, foi mediado pelo facilitador, que inicialmente buscou a expressão verbal dos participantes que estiveram em cena e, posteriormente, a expressão dos demais membros do grupo.

Esse momento oportunizou ao enfermeiro voluntário a reflexão acerca da assistência de enfermagem que ele prestou, considerando a avaliação do contexto do parto, fatores de risco, julgamento clínico, estimativa visual de perda sanguínea, cálculo do índice de choque, diagnóstico precoce da HPP, chamar ajuda e iniciar o protocolo medicamentoso para tratamento da HPP. Além disso, foram discutidos os pontos positivos e os pontos de melhoria.

### Análise dos resultados e estatística

Os dados foram compilados e analisados através do software R, versão 4.2.0. Foram utilizadas medidas de tendência central e dispersão. O teste de Shapiro-Wilk foi aplicado para verificar a suposição de normalidade. Para comparar os dois grupos, GC e GI, conforme as pontuações em cada instrumento com as demais variáveis, foi aplicado o teste de Wilcoxon. Foram formuladas as seguintes hipóteses: a hipótese nula (H₀) afirma que não há diferença entre a simulação clínica e o método tradicional no conhecimento do enfermeiro para o manejo da HPP; a hipótese alternativa (H₁) sugere que existe diferença no conhecimento do enfermeiro entre os dois métodos. Para este teste, considerou-se significância estatística quando p < 0,05.

## RESULTADOS

Dos 41 enfermeiros randomizados para a amostra, 37 permaneceram até o final do estudo, sendo que 18 compuseram no GI e 19 o GC. Foi homogênea a distribuição entre profissionais do GI e GC. O tempo médio de atuação no setor foi de 8 anos, embora estivessem formados há, aproximadamente, 16 anos.

A maioria dos enfermeiros era mulheres, na faixa etária entre 31 e 40 anos, com tempo de formação em enfermagem entre 2 e 36 anos, com variáveis níveis de pós-graduação, com especialidades em áreas diversas, além de, mestres e doutores (Tabela 1).

**Tabela 1:** Distribuição das variáveis relacionadas às características dos participantes e perfil dos enfermeiros que atuam nos setores de assistência ao pré-parto, parto e puerpério, Fortaleza, Ceará, Brasil, 2023

Variáveis sociodemográficas	Grupo Controle		Grupo Intervenção		Total	
	n=19	%	n=18	%	N=37	%
Sexo						
Feminino	18	48,7	11	29,7	29	78,4
Masculino	2	5,4	6	16,2	8	21,6
Faixa etária						
31-40 anos	9	24,3	11	29,7	20	54,0
41-50 anos	7	18,9	5	13,5	12	32,4
51-60 anos	3	8,1	2	5,4	5	13,5
Nível de formação						
Graduação	---	---	1	2,7	1	2,7
Especialização	15	40,5	11	29,7	26	70,2
Mestrado	4	10,8	4	10,8	8	21,6
Doutorado	---	---	2	5,4	2	5,4
Esp. Enfermagem Obstétrica						
Sim	8	21,6	11	29,7	19	51,3
Não	11	29,7	7	18,9	18	48,6
Setor de atuação						
Emergência	2	5,4	5	13,5	7	18,9
UTI Materna	2	5,4	---	---	2	5,4
Centro Cirúrgico	4	10,8	2	5,4	6	16,2
Clínica Cirúrgica	2	5,4	1	2,7	3	8,1
Clínica Obstétrica	4	10,8	3	8,1	7	18,9
Centro Obstétrico	5	13,5	5	13,5	10	27,1
SRPA	---	---	2	5,4	2	5,4
Tempo de atuação no setor						
< 6 meses	---	---	2	5,4	2	5,4
1-10 anos	15	40,5	13	35,1	28	75,6
11-20 anos	3	8,1	3	8,1	6	16,2
21-30 anos	1	2,7	---	---	1	2,7
Total	19	51,3	18	48,7	37	100

*UTI - Unidade de Terapia Intensiva; SRPA - Sala de Recuperação Pós-Anestésica.*

Na linha de base dos enfermeiros, a maioria apresentava, pelo menos, uma experiência prévia com simulação; mais de 80% dos enfermeiros receberam algum tipo de treinamento prévio para manejo da HPP e, que, a quase totalidade dos participantes, tiveram essa oportunidade ofertada pela própria instituição que trabalham. Para a maioria deles, o treinamento para manejo da HPP havia sido ministrado há mais de um ano. Cerca de 91% dos enfermeiros referiram já ter vivenciado a assistência a uma puérpera com HPP, entretanto, aproximadamente, 30% dos enfermeiros afirmaram que não se sentia preparado para o manejo da HPP, por medo/insegurança ou ainda falta de treinamento/conhecimento (Tabela 2).

**Tabela 2:** Distribuição das variáveis relacionadas a participação dos enfermeiros em atividades envolvendo simulação e treinamento para manejo da hemorragia pós-parto, Fortaleza, Ceará, Brasil, 2023

Atividades envolvendo simulação e treinamento para manejo da HPP	Grupo Controle		Grupo Intervenção		Total	
	n=19	%	n=18	%	N=37	%
Experiência prévia com simulação (n=37)						
Sim	10	27,0	11	29,7	21	56,7
Não	9	24,3	7	19,0	16	43,3
Treinamento prévio para manejo da HPP (n=37)						
Sim	15	40,5	16	43,3	31	83,8
Não	4	10,8	2	5,4	6	16,2
Em qual contexto (n=31)						
Curso promovido pela instituição	14	45,2	16	51,6	30	96,8
Em contextos diversos	1	3,2	---	---	1	3,2
Tempo do último treinamento (n=31)						
< 1 ano	2	6,5	4	13,0	6	19,5
1-2 anos	11	35,5	7	22,5	18	58,0
>2 anos	2	6,4	5	16,1	7	22,5
Já manejou uma HPP (n=37)						
Sim	18	48,6	16	43,3	34	91,9
Não	1	2,7	2	5,4	3	8,1
Em qual contexto (n=34)						
Em cursos de aprimoramento	2	5,9	2	5,9	4	11,8
Durante o plantão	15	44,1	13	38,2	28	82,3
Em contextos diversos						
Sente-se preparado para o manejo da HPP (n=37)						
Sim	15	40,5	11	29,7	26	70,2
Não	4	10,8	7	19,0	11	29,8
Se não quais os motivos (n=11)						
Medo e/ou insegurança	2	18,2	2	18,2	4	36,4
Falta de equipe treinada/conhecimento	2	18,2	5	45,4	7	63,6
Total	19	51,3	18	48,7	37	100

*HPP - Hemorragia Pós-Parto.*

Quanto a comparação entre o número de acertos após a intervenção e a competência clínica dos enfermeiros, observou-se que houve significância estatística (*p*<0,00), demonstrando associação entre o nível de competência prévia e desempenho cognitivo dos participantes após o cenário de simulação. Dessa forma, demonstra-se que houve aumento/melhoria do conhecimento acerca do manejo da HPP, com um percentual maior que 60% no número de acertos no pós-teste.

Houve melhoria em relação ao número de acertos antes e depois da intervenção. No pré-teste, dentre as 15 questões objetivas, a média de acertos foi de 3,8 e um intervalo de 1-9 acertos, sendo que nenhum enfermeiro conseguiu acertar mais que 60% das questões apresentadas no pré-teste. No pós-teste, das 15 questões apresentadas, a média de acertos foi de 13,24, com intervalo de 9-15 acertos, com mínimo de 60% e máximo de 100% de desempenho cognitivo. Observa-se um resultado estatisticamente significante (*p*=0,00) para os resultados pós-intervenção (Tabela 3).

**Tabela 3:** Distribuição do número de acertos no pré-teste e o número de acertos no pós-teste. Resultados antes e depois da intervenção, Fortaleza, Ceará, Brasil, 2023

Questionamentos		Acertos Pré-teste	Acertos Pós-teste	Valor de *p*
1	Em relação as possíveis causas de HPP	1/37	23/37	0,00^*^
2	Acerca do tratamento precoce da HPP	0/37	36/37	
3	Acerca da Estratificação de Risco para a HPP	4/37	31/37	
4	Avaliação inicial frente a uma paciente com HPP	13/37	36/37	
5	Com relação a Hora de Ouro na HPP	8/37	32/37	
6	Referentes ao Índice de Choque (IC)	5/37	34/37	
7	Acerca do diagnóstico visual de perda sanguínea na HPP	1/37	27/37	
8	Acerca da prescrição padrão para o manejo da HPP	6/37	29/37	
9	Acerca do trabalho em equipe para o manejo de HPP	11/37	37/37	
10	Acerca do manejo não-cirúrgico da HPP	10/37	31/37	
11	Em relação às medidas de prevenção da HPP	6/37	36/37	
12	Acerca do método clínico para diagnóstico da HPP	2/37	36/37	
13	Competências do Enfermeiro frente a um quadro de HPP	15/37	36/37	
14	Acerca do material/kit de HPP	7/37	36/37	
15	Definição de HPP maciça	4/37	30/37	

* *Teste de Wilcoxon. HPP - Hemorragia Pós-Parto.*

Os resultados dos enfermeiros que integraram o GC, expostos à aula expositiva e ao treinamento habitualmente ofertado pela instituição, observou-se melhoria do desempenho cognitivo em 57,4%, quanto às questões relacionadas ao conhecimento do manejo da HPP antes e depois. Quanto aos resultados do GI, expostos à aula expositiva e ao cenário de simulação clínica, pode-se observar que a melhoria do desempenho cognitivo em 67,8%.

Na Tabela 4 foram representadas as medidas de tendência central (média e mediana) e as medida de dispersão (desvio padrão) do pré-teste e pós-teste de cada grupo. Além da análise comparativa intergrupo, antes e depois, observou-se diferença estatística (*p*<0,00), rejeitando a hipótese nula e indicando que a simulação clínica tem um efeito significativo no conhecimento dos profissionais de enfermagem em comparação ao método tradicional.

**Tabela 4:** Medidas relacionadas ao conhecimento antes (pré-teste) e depois (pós-teste) entre os grupos controle e intervenção, Fortaleza, Ceará, Brasil, 2023

Grupos		Média	Mediana	DP	Valor de *p*
Pré-teste	Grupo Controle	4,05	4	1,87	0,00^*^
	Grupo Intervenção	3,72	3	2,22	
Pós-teste	Grupo Controle	12,6	13	1,68	
	Grupo Intervenção	13,9	14	1,21	

DP - desvio padrão;

* Teste de Wilcoxon.

## DISCUSSÃO

Quanto aos resultados da *baseline*, a experiência prévia em simulação mostrou que a maioria dos enfermeiros referiu ter tido contato com algum tipo de ensino utilizando simulação. De fato, as experiências de aprendizagem simuladas podem proporcionar aos participantes o desenvolvimento de habilidades clínicas específicas, além de apoiar a tomada de decisão e o desenvolvimento de habilidades de pensamento crítico em um ambiente seguro e controlado por meio de cenários de aprendizagem simulados^(13)^. A natureza interativa e multissensorial de um cenário de aprendizagem simulado cria um ambiente no qual os participantes têm a oportunidade de receber feedback imediato do facilitador acerca de seu desempenho^(14)^.

No que diz respeito a essa ferramenta de integração teórico-prática, a simulação clínica oferece contribuições como: possibilidade de treinamento prévio ao contato com o paciente real; oportunidade para o desenvolvimento e aplicação de conhecimentos e habilidades em um ambiente seguro; minimização das chances de erros mediante repetição e treinamento exaustivo pelo discente; aumento da percepção da responsabilidade frente ao cuidado; experiência valiosa para a aprendizagem psicomotora e desenvolvimento de pensamento crítico-reflexivo; aumento da confiança e habilidades comunicativas; e ampliação da aprendizagem contextualizada, transversal à integralidade do cuidado^(15)^.

O uso da simulação como ferramenta para o processo de ensino-aprendizagem, em estudos realizados nos Estados Unidos e na Índia, mostrou que a utilização de simuladores para o treinamento da equipe pode fornecer uma referência para cultivar a capacidade de cooperação interprofissional, melhorar o desempenho clínico no manejo da HPP, aperfeiçoar a comunicação e a adesão ao protocolo dos participantes durante cenários simulados de HPP, contribuindo para a redução da morbidade por essa emergência obstétrica^(10,16)^.

O conhecimento dos enfermeiros examinados, tanto no GC quanto no GI mostrou-se semelhante no pré-teste e apresentou melhora na avaliação pós-intervenção. Conjuntamente aos dados descritos, um estudo de intervenção, do tipo antes e depois, realizado com 46 estudantes de enfermagem, corroborou com os resultados aqui apresentados, demonstrando que a aprendizagem a partir da simulação clínica conferiu *scores* de conhecimento maiores comparados àqueles adquiridos por meio de aula expositiva com demonstração de habilidades^(17)^. Concernente aos nossos achados, estudos de metanálise têm demonstrado que a educação em enfermagem baseada em simulação é um método eficaz no processo de ensino-aprendizagem^(18)^.

Destaca-se a melhoria no desempenho cognitivo que a utilização da simulação clínica promove, apresentando potenciais impactos positivos na aprendizagem e prática clínica dos enfermeiros. Aclarado o grande potencial da simulação para tratar de problemas relacionados ao desenvolvimento de conhecimentos e competências dos profissionais, na medida em que supera o processo de recepção passiva centrado na transmissão de conteúdos presente na formação^(19)^.

Ambos os grupos expressaram haver outras questões relacionadas à aprendizagem significativa que possam estar interferindo nos resultados e impedindo o enfermeiro de sentir-se preparado para atender uma puérpera com hemorragia. Como revelado por uma revisão integrativa, que objetivou sintetizar a produção científica acerca da Teoria da Aprendizagem Significativa no processo de ensino-aprendizagem em Enfermagem, os autores concluíram que é necessário romper com a dicotomia entre teoria e prática e promover a articulação dos conteúdos com a ação, introduzindo o discente como autor de seu próprio conhecimento^(20)^.

Um estudo descritivo e teórico, de abordagem qualitativa, relatou em seus achados que articular a Teoria da Aprendizagem Significativa e Andragogia é possível, principalmente quando lidamos com estudantes adultos. Porém, antes de qualquer tentativa de colocar em prática essa articulação, os docentes-facilitadores devem sair do papel de transmissores de conhecimento e os estudantes devem abandonar o papel de receptores passivos. Trata-se de um exercício difícil, mas que deve ser praticado continuamente, pois só assim as atividades de ensinar e aprender serão transformadas em momentos de satisfação e, com isso, haverá maior apropriação dos objetivos propostos^(20)^.

A respeito da prática experiencial, os resultados de um estudo de abordagem qualitativa e descritiva, realizado com alunas do curso de especialização em enfermagem obstétrica, revelaram que a utilização de simuladores de pacientes humanos de alta fidelidade pode ser usada em um cenário de caso complexo para promover a aprendizagem experiencial de uma emergência clínica^(14)^.

Além disso, no âmbito da prática clínica, a realização de abordagens educativas que considerem as experiências práticas possui um caráter substancial, uma vez que o profissional constrói suas próprias estruturas cognitivas e, a partir da interação com o meio, consolida o conhecimento^(21)^.

A execução de habilidades específicas é fundamental, mas é com a resolução de cenários complexos, em contextos simulados, que os enfermeiros consolidam seus saberes e desenvolvem capacidades de raciocínio crítico, julgamento e tomada de decisão, além de competências nas diferentes dimensões técnica, atitudinal e ética^(22)^.

Benner, em sua última obra, faz um apelo por uma transformação radical na educação de enfermagem, na qual os alunos devem desenvolver uma relação fluida entre conhecimento científico, *know-how* qualificado e ética, infundindo o currículo com aprendizado experiencial rico. A aprendizagem experiencial deve ocorrer não apenas no ambiente clínico, mas também na sala de aula e no laboratório de habilidades. Esse aprendizado deve ajudar os alunos a desenvolver um senso de relevância, raciocínio clínico e imaginação clínica enquanto formam sua identidade como enfermeiros^(23)^.

### Limitações do estudo

Como limitações do estudo, destacam-se a impossibilidade de avaliar a retenção de conhecimento a longo prazo, pois os resultados não foram acompanhados ao longo do tempo, e o fato de ter sido realizado em uma única instituição. Além disso, houve um número amostral reduzido devido à dificuldade de participação dos enfermeiros, que precisaram conciliar sua carga de trabalho com a intervenção educativa, e por perdas amostrais. O estudo também foi limitado a um único ambiente, o centro obstétrico.

### Contribuições para a Área

A HPP é uma condição altamente evitável de morbidade e mortalidade materna em todo o mundo. Os esforços para melhorar o diagnóstico e o gerenciamento da HPP podem incluir a simulação clínica. A contribuição do estudo refere-se à possibilidade de melhoria da aprendizagem significativa no manejo da HPP, junto à aquisição de competências por enfermeiros e profissionais de saúde. A presente investigação contribui para o avanço científico de pesquisas sobre a simulação em enfermagem, por meio da utilização de cenários.

Com isso, o estudo apresenta uma potente ferramenta para a melhoria da qualidade da assistência nessa emergência obstétrica e lança um olhar sobre uma situação que, infelizmente, ainda ceifa a vida de tantas mulheres e que necessita de tomada de decisão dos enfermeiros para mudar essa realidade.

## CONCLUSÕES

A intervenção educativa com a utilização da simulação clínica como estratégia de ensino-aprendizagem mostrou-se eficaz para a aquisição de conhecimento pelos enfermeiros sobre o manejo da HPP e, dessa forma, proporcionou melhorias nesses conhecimentos, fundamentais para o alcance do ODS 3.1, contribuindo para a redução da mortalidade materna. Os resultados revelaram um impacto significativo no desempenho cognitivo dos enfermeiros, independentemente do nível de formação e experiência prévia, demonstrado pela avaliação após a atividade de simulação comparada ao período pré-intervenção.
